# Discrete States of a Protein Interaction Network Govern Interphase and Mitotic Microtubule Dynamics

**DOI:** 10.1371/journal.pbio.0050029

**Published:** 2007-01-16

**Authors:** Philipp Niethammer, Iva Kronja, Stefanie Kandels-Lewis, Sonja Rybina, Philippe Bastiaens, Eric Karsenti

**Affiliations:** 1 Cell Biology and Biophysics Department, European Molecular Biology Laboratory, Heidelberg, Germany; 2 Department of Systems Biology, Harvard Medical School, Boston, Massachusetts, United States of America; National Cancer Institute-National Institutes of Health, United States of America

## Abstract

The cytoplasm of eukaryotic cells is thought to adopt discrete “states” corresponding to different steady states of protein networks that govern changes in subcellular organization. For example, in *Xenopus* eggs, the interphase to mitosis transition is induced solely by activation of cyclin-dependent kinase 1 (CDK1) that phosphorylates many proteins leading to a reorganization of the nucleus and assembly of the mitotic spindle. Among these changes, the large array of stable microtubules that exists in interphase is replaced by short, highly dynamic microtubules in metaphase. Using a new visual immunoprecipitation assay that quantifies pairwise protein interactions in a non-perturbing manner in *Xenopus* egg extracts, we reveal the existence of a network of interactions between a series of microtubule-associated proteins (MAPs). In interphase, tubulin interacts with XMAP215, which is itself interacting with XKCM1, which connects to APC, EB1, and CLIP170. In mitosis, tubulin interacts with XMAP215, which is connected to EB1. We show that in interphase, microtubules are stable because the catastrophe-promoting activity of XKCM1 is inhibited by its interactions with the other MAPs. In mitosis, microtubules are short and dynamic because XKCM1 is free and has a strong destabilizing activity. In this case, the interaction of XMAP215 with EB1 is required to counteract the strong activity of XKCM1. This provides the beginning of a biochemical description of the notion of “cytoplasmic states” regarding the microtubule system.

## Author Summary

When eukaryotic cells undergo cell division, a dramatic reorganization occurs during the transition from interphase to metaphase. The cell rounds up, chromosomes condense, the nuclear envelope breaks down, and microtubules (proteins that help maintain the cell's shape) become very short and dynamic before assembly of the mitotic spindle (the structure that pulls chromosomes apart). Although it is known that the CDK1 kinase induces this reorganization, the precise mechanisms that regulate such coordinated changes are not yet understood. To investigate the regulation of microtubule dynamics, we applied a new method, called visual immunoprecipitation (VIP), that enables simultaneous visualization of multiple protein interactions in cell extracts. There are two known major regulators of microtubule dynamics: a stabilizer (XMAP215) and a destabilizer (XKCM1); a series of other molecules (EB1, APC, and CLIP 170) are also involved, although their roles in the global regulation of microtubule dynamic instability are not as clear. We show here that microtubules are stable during interphase because the destabilizer is inhibited by the other molecules. During mitosis, however, the destabilizer is released, triggering the alteration of microtubule structure and dynamics. Thus, microtubule dynamics change in response to a dramatic switch in the interactions of a set of proteins.

## Abstract 3

Previously unknown interactions between different microtubule-associated proteins (MAPs) are identified via a new technique, termed visual immunoprecipitation.

## Introduction

Understanding the functional consequences of interactions between multiple components of a complex biological system is a challenge. Typical proteomic approaches provide only snapshots of one specific state of a network of interactions. Usually, they do not contain information about how biochemical changes cause functional changes, nor do they reveal the extent to which protein interactions occur in the cell [1]. By contrast, typical cell biological studies concentrate on the function of only one component or interaction at a time, mostly by using RNA interference (RNAi), genetics, co-localization, or immunodepletion experiments. Those approaches tend to overemphasize the importance of binary interactions while missing the significance of network behavior.

Several MAPs involved in the regulation of microtubule (MT) dynamics in vivo have been characterized recently (XMAP215, XKCM1, EB1, APC, and CLIP170) [2]. XMAP215 and EB1 are known MT stabilizers, whereas XKCM1 is a destabilizer [3–5]. The function of APC remains unclear although several reports indicate that it possibly interacts with EB1 in order to stabilize MTs [6,7]. CLIP170 seems to participate in the local regulation of MT dynamics [8–10].

Although there is a wealth of data on these MAPs, no coherent picture concerning their potential collective effects on MT growth in one single system has emerged yet. Thus, we decided to examine the mutual interactions of the above-mentioned molecules and their effects on MT dynamics in *Xenopus* egg extracts. This undiluted frog egg cytoplasm recapitulates the cell cycle, mimics in vivo conditions, and allows biochemical analysis of MT dynamics in two well-defined states, interphase and mitosis [11–13]. Using a newly developed interaction assay (visual immunoprecipitation [VIP]) we have imaged directly in such extracts the interaction patterns of MAPs in interphase and mitosis. We have correlated this study with an analysis of MT growth in response to immunodepletion of individual MAPs as a function of cell-cycle state. The combined data of these two approaches strongly suggest that in interphase, the extensive growth and stability of MTs are caused by an inhibition of the destabilizing factor XKCM1 through multiple interactions with other MAPs (XMAP215, CLIP170, EB1, and APC). By contrast, in mitosis, MTs become highly dynamic because these interactions are disrupted, activating the catastrophe-promoting activity of XKCM1. Under such conditions, a complex of XMAP215 and EB1 is required for MT growth.

## Results

### The Visual Immunoprecipitation Assay

Standard immunoprecipitation (IP) techniques are time consuming and insensitive to low-affinity interactions, and their results vary considerably with different washing conditions. Moreover, interactions that are maintained by enzymatic activities in the cytoplasm are likely to be lost during the IP procedure. Such approaches are, therefore, not well adapted to the analysis of protein interaction networks that are likely to consist of functionally important low-affinity or labile interactions. Most of the in situ microscopy techniques currently available (fluorescence resonance energy transfer [FRET] and fluorescence cross-correlation spectroscopy [FCCS]) require advanced instrumentation, elaborate image analysis, and fluorescent labeling of all interaction partners that are to be studied. In addition, they are not well suited for the simultaneous detection of multiple interactions in the same sample. Thus, we set out to develop VIP as a novel microscopy technique allowing direct imaging of multiple protein interactions in *Xenopus* egg extracts.

**Figure 1:**
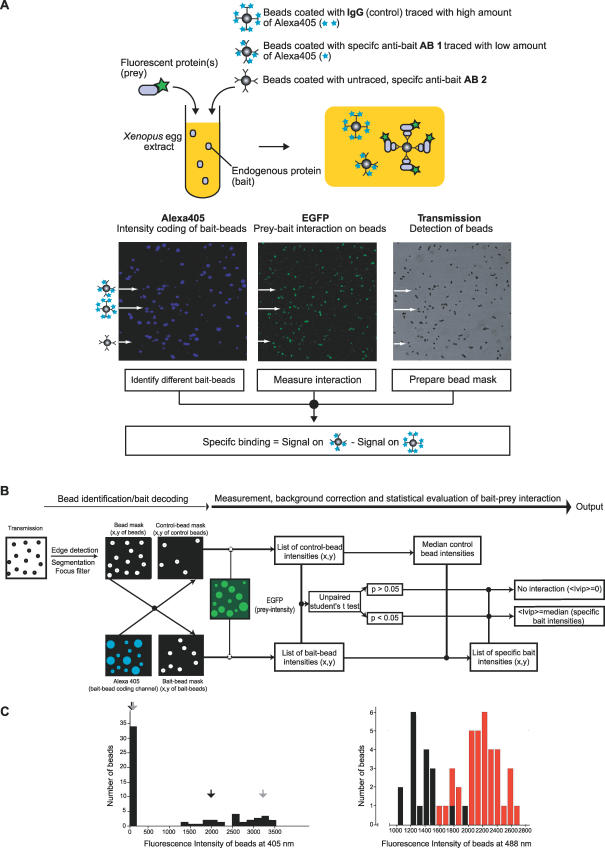
The VIP Assay (A) Scheme of VIP methodology. AB1/AB2: specific antibodies recognizing endogenous bait. (B) Flow diagram of the automated image processing used for VIP. (C) Histograms of Alexa405 fluorescence (left panel) and EGFP fluorescence (right panel) on beads in an in vitro VIP experiment in which 1,600 nM EB1-EGFP was titrated into CSFXB buffer containing 300 nM XMAP215, anti-XMAP215 beads, and Alexa405-IgG beads. Different bead species (IgG/bait beads) are encoded by distinct Alexa405 emission (left panel). Each bead species is defined by the S.D. of Gaussian distributions (the black arrow marks the beginning and the grey arrow the end of the confidence interval) fitted to the Alexa405 histogram. Beads with Alexa405 intensities lying within the S.D. of the low-intensity population are defined as IgG beads. Beads with Alexa405 intensities lying within the S.D. of the high-intensity population are defined as bait beads (here: anti-XMAP215 beads). The emission of the two species (black: IgG beads; and red: anti-XMAP215 beads) in the EGFP channel is represented as a histogram (right panel). The VIP signal is calculated by subtracting the median intensity of bait beads in the EGFP channel by the median intensity of IgG beads in the EGFP channel only if there is a significant difference (*p* < 0.05, unpaired *t*-test) between these two populations.

Antibody-coated, micrometer-sized beads are introduced into extracts that contain fluorescently labeled recombinant proteins. Interaction between an endogenous protein trapped on the bead (bait) and a labeled protein (prey) in the extract is detected by the fluorescence signal of the prey bound to the beads (Figure 1A). By encrypting the different bait-bead loads with distinguishable fluorescence intensities at a given spectral window, multiple protein interactions can be detected simultaneously within the same sample (Figure 1A). The specific signals are corrected for varying contributions of autofluorescence or unspecific binding, measured on Alexa405-labeled IgG-coated control beads included within the same sample (Figure 1B). Images are acquired with a confocal microscope. Confocal imaging allows optimization of the ratio between background and bead fluorescence by adjusting the pinhole/slice thickness. Sequential line scanning of all channels ensures minimal bleed through or cross-excitation. Acquisitions include a transmission and Alexa405 image, together with a variable number of images from “signal” channels (prey emission, e.g., enhanced green fluorescent protein [EGFP]; Figure 1A and 1B). The transmission image allows the identification of single beads by edge detection (Figure 1B). Then, coordinates *(x,y)* are assigned to that bead by segmentation (Figure 1B). Detection of beads from the transmission image has the advantage that object recognition is not biased by a (potentially heterogeneous) fluorescence signal coming from the bead. Unfocused beads are excluded from the bead mask on reflection criteria. Control and bait beads are subsequently distinguished as different intensity populations (Figure 1C; Alexa405, 405-nm histogram) in the Alexa405 channel (bait-coding channel). This allows the generation of two bead masks, one comprising only bait beads, the other comprising only control beads. The binding of the fluorescent prey to the bait beads (bait-bead intensity) and to control beads (control-bead intensities) is then measured by applying the masks on the “prey” channels, e.g., EGFP channel (Figure1C; EGFP, 488-nm histogram). Specific bait-bead intensities are calculated by subtracting the median control-bead intensity from the fluorescence intensity on each bait bead in the same image. The specific bait intensity in each image is calculated as the median of the specific bait-bead intensities (<Ivip>) only if the intensity difference of bait and control beads in an image is found to be significant (*p* < 0.05) by an unpaired Student *t*-test. Otherwise (*p* > 0.05), the signal is set to zero (Figure 1B). The Student *t*-test filter ensures that signals on the bait beads are only scored as interactions if there is a significant intensity difference with the control beads in the same image. Background subtraction allows a direct comparison of interaction signals between samples from different extracts. The image processing has been automated using MATLAB routines (http://www.mathworks.com/).

**Figure 2:**
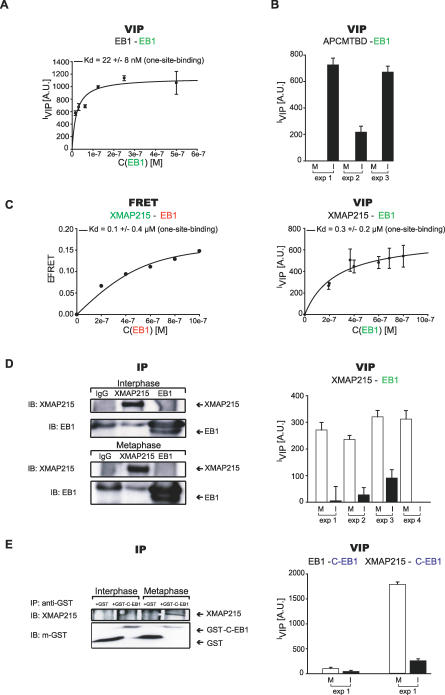
Validation of VIP Assay (A) VIP binding isotherm of EB1-EGFP to αEB1-antibody interaction fitted with a one site–binding model. A.U., arbitrary units. (B) Interaction of EB1-EGFP (prey: EGFP protein added to the extract) with APCMTBD (bait: recombinant protein added to the extract that binds to the anti-APC–coated beads) in metaphase (M, open bars) and interphase (I, filled bars). The three pairs of bars correspond to three independent experiments carried out in different extracts. A.U., arbitrary units; exp, experiment. (C) FRET binding isotherm of recombinant XMAP215-EGFP to recombinant EB1Cy3 in CSFXB buffer (left panel) and the VIP binding isotherm of recombinant XMAP215 to EB1-EGFP in CSFXB buffer (right panel). Both isotherms are fitted with one site–binding models. A.U., arbitrary units. (D) IP of XMAP215 or EB1 (left panel) and XMAP215/EB1-EGFP VIP (right panel) in interphase and metaphase extracts. Co-precipitation of EB1 or XMAP215 in IP experiments was probed by SDS-PAGE/immunoblotting with the respective antibodies (immunoblot [IB] with XMAP215 antibody and with EB1 antibody). In the VIP experiment, endogenous XMAP215 served as bait and EB1-EGFP as fluorescent prey. In the right panel, the four pairs of bars correspond to four independent experiments performed in metaphase (M, open bars) and interphase (I, filled bars) extracts. A.U., arbitrary units; exp, experiment. (E) Left panel shows IP pull down of 2 μM GST and GST-C-EB1 using apolyclonal αGST antibody. Co-precipitation of XMAP215 was probed by SDS-PAGE/immunoblotting with the XMAP215 antibody (IB: XMAP215). Immunoblot (IB) with monoclonal αGST antibody (m-GST) shows that GST and GST-C-EB1 were added to interphase and metaphase extracts accordingly. Right panel shows the VIP experiment of EB1-GST-C-EB1Cy5 and XMAP215-GST-C-EB1Cy5 in metaphase (M, open bars) and interphase (I, filled bars) extracts. Error bars represent the s.e.m. of <I_VIP_> derived from at least three different microscopic fields. A.U., arbitrary units, exp, experiment.

To validate the VIP assay, we measured the dissociation constant (Kd) of an antibody-antigen interaction and confirmed the already reported binding of EB1 to the MT-binding domain of APC (APCMTBD; Figure S1A) [14]. As anticipated, VIP revealed a nanomolar Kd for the antibody-antigen interaction (Figure 2A) and gave a strong signal for the APCMTBD-EB1 interaction specifically in interphase (Figure 2B), but not in metaphase in which the signal was below the significance criteria. We also examined the interaction in buffer (CSF-XB) of purified XMAP215 with EB1-EGFP using VIP and of XMAP215-EGFP with EB1-Cy3 using FRET. The two methods gave similar Kds (Figure 2C). Although we could detect the interaction of the endogenous XMAP215 and EB1 in metaphase extracts only by VIP (Figure 2D, right panel) and not by regular IP (Figure 2D, left panel), the binding of XMAP215 to the C-terminal domain of EB1 (C-EB1; Figure 2E) was revealed by both techniques.

### MAP Interaction Networks in Interphase and Mitosis

**Figure 3:**
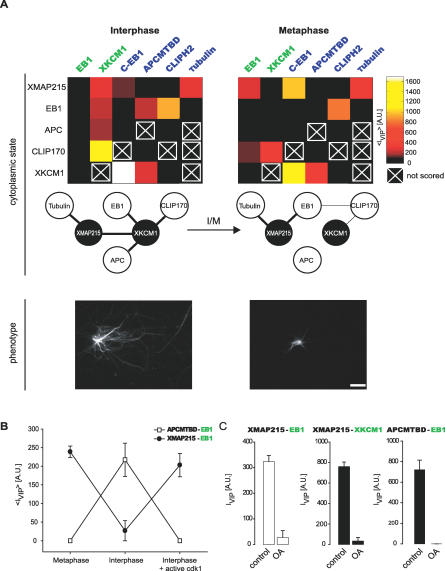
Change in the MAP Interaction Network at Interphase/Metaphase Transition (A) Pairwise interaction of MAPs in interphase and metaphase are represented as color-coded tables (upper panel). The color code corresponds to <I_VIP_> of at least three different microscopic fields. Proteins in green are EGFP tagged, and those in blue are Cy5 labeled. Below each table is a schematic representation of the network states in interphase and metaphase. Line thickness corresponds to interaction strength normalized to the strongest signal detected in interphase or metaphase. In the lower panel, an interphase aster is shown on the left and a mitotic one on the right (B) CDK1 activity induced a change in EB1-APCMTBD and EB1-XMAP215 interactions. EB1-EGFP fluorescence was measured simultaneously on beads coated with anti-XMAP215 and anti-APCMTBD antibodies in the presence of APCMTBD, and added to metaphase, interphase, and interphase extracts after addition of active, purified CDK1. Error bars represent the s.e.m. of <I_vip_> measured in six different microscopic fields. A.U., arbitrary units. (C) XMAP215/EB1-EGFP interaction in metaphase (open bars) and XMAP215/XKCM1-EGFP and APCMTBD/EB1-EGFP interaction in interphase (filled bars) before (control) and after incubation with 10 μM okadaic acid (OA). Error bars represent the s.e.m. of <I_VIP_> derived from at least three different microscopic fields.

Using VIP, we set out to visualize the interaction pattern between five important regulators of MT dynamics (XMAP215, XKCM1, CLIP170, APC, and EB1) in interphase and metaphase *Xenopus* egg extracts. We observed a switch in global MAP interactions between interphase and metaphase (Figure 3A). First, we found a clear interaction between the MT destabilizer XKCM1 and all other MAPs in interphase that disappeared or was strongly decreased in metaphase. Second, the MT stabilizer XMAP215 that did not interact with EB1 in interphase was found to do so in metaphase. Third, CLIP170 that did not interact with EB1 in interphase did so weakly in metaphase. XMAP215-EB1 and XMAP215-XKCM1 binding persisted upon switching of bait and prey (unpublished data). Interestingly, XMAP215, but not EB1, showed a strong interaction with tubulin both in interphase and metaphase. This is in line with a recent study that reported an interaction of the yeast homolog of XMAP215 (Stu2p) with tubulin [15]. With an anti-APCMTBD antibody, we could not detect a cytosolic interaction between full-length APC and full-length EB1-EGFP either in interphase or metaphase (Figure 3A). With the same bait-prey combination, we found that the 16 nM of added recombinant APCMTBD did interact with EB1-EGFP specifically in interphase (unpublished data) as previously reported in the literature [7]. Additionally, we found that endogenous EB1 recruited to an anti-EB1 antibody interacted with Cy5-labeled APCMTBD also only in interphase (Figure 3A). Thus, antibody interference with bait-prey binding cannot explain the lack of interaction between full-length APC and EB1. This also indicates that the APC fragment missing in the APCMTBD contributes to the regulation of interaction between full-length APC and EB1 under physiological conditions. By contrast, antibody interference seemed to be an issue for the XKCM1-EB1 interaction. Here, we could only reveal the interaction by using endogenous EB1 as bait and XKCM1-EGFP (Figure S1B) as prey. Because full or partial overlap of bait-binding epitopes for antibody and prey is anticipated to interfere with bait-prey interaction, for VIP, as well as for other antibody-based methods, confidence about negative signals absolutely requires positive controls. In the present case, the strong differences in MAP interactions observed in interphase and metaphase extracts provide positive and negative internal controls. Interestingly, the C-EB1 fragment interacted with XMAP215 in metaphase and to a lesser extent in interphase, suggesting that the regulated interaction observed between the wild-type proteins was partly removed from the fragments. XKCM1 that interacted strongly with C-EB1 and the APCMTBD in interphase still did so in metaphase, showing that cell-cycle regulation observed for wild-type proteins was also lost in these fragments. A similar observation was made for the interaction of EB1 with a fragment of CLIP170 (Figure 3A; the CLIP-170 H2 domain [CLIPH2], Figure S1A).

To determine whether the observed switch in MAP interaction was triggered by cyclin-dependent kinase 1 (CDK1), we followed the interaction between XMAP215, EB1, and the APCMTBD when the extracts were induced to shift from metaphase to interphase by adding calcium and back to metaphase by adding purified active CDK1/cyclinB complex [16]. As shown in Figure 3B, the interaction between XMAP215 and EB1 as well as between APCMTBD and EB1 changed accordingly, demonstrating that these interactions are indeed modulated by CDK1 activity. Importantly, XMAP215-EB1, XMAP215-XKCM1, and APCMTBD-EB1 binding were abolished by addition of okadaic acid (OA) to the extracts (Figure 3C), indicating that their steady-state interaction levels are also maintained by phosphatase activities present in the extract.

### Causal Relationships between the MAP Network and MT Dynamic States

**Figure 4:**
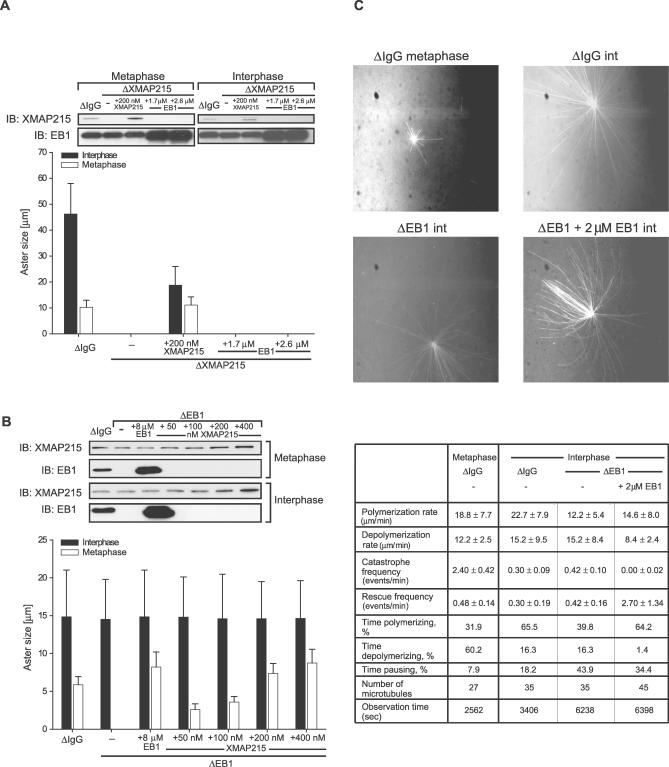
MT Stabilizing Activities of EB1 and XMAP215 in Interphase and Metaphase *Xenopus* Egg Extracts (A) Average size of asters in response to depletion of XMAP215 (ΔXMAP215) from the extract and the addition of recombinant EB1 to the ΔXMAP215 extract. Error bars represent the S.D. (*n* > 30). Upper panel: immunoblot (IB) of MAPs after indicated treatments. (B) Average size of asters in response to depletion of EB1 (ΔEB1) from the extract and the addition of recombinant XMAP215 to the ΔEB1 extract. Error bars represent the S.D. (*n* > 30). Upper panel shows the immunoblot (IB) of MAPs after the indicated treatments. (C) Still images of MT asters after the indicated treatments are shown in the upper panel. Table summarizing the dynamic parameters of MT growth in response to EB1 depletion (ΔEB1) and add back of recombinant EB1 into ΔEB1 interphase extracts is shown in the lower panel.

The two discrete MAP interaction states correlate with two discrete phenotypes of the MT system (Figure 3A, lower panel). In interphase extracts, MTs are long and stable because there are almost no catastrophes and they grow continuously as long as there is enough soluble tubulin and MAPs. In mitosis, catastrophes are relatively frequent, resulting in highly dynamic MTs that reach a steady-state length after a few minutes of growth [17]. In an attempt to establish a causal relationship between the observed MAP network states and these MT phenotypes, we performed a variety of perturbation assays and analyzed their effects on the length of MTs nucleated from centrosomes added to egg extracts. Depletion of XMAP215 completely blocked MT growth both in interphase and metaphase extracts as previously reported (Figure 4A, Figure S2A) [3]. Adding back XMAP215 restored MT growth. EB1 depletion dramatically reduced MT growth in metaphase, but caused only a minor reduction in aster size in interphase (Figure 4B, Figure S2B) [4]. When MT dynamics were measured in interphase, the effect of EB1 depletion was a 2-fold decrease in polymerization rate, associated with a 2-fold increase in pausing time (Figure 4C). These dynamic measurements are consistent with the small effect of EB1 depletion on MT length in interphase (Figure 4B). Adding back recombinant EB1 to EB1-depleted metaphase extracts restored MT growth (Figure 4B, Figure S2B). Interestingly, the inhibition of MT growth caused by EB1 depletion from metaphase extracts could also be rescued by adding back an excess of XMAP215 (Figure 4B). On the other hand, adding back only EB1 to XMAP215-depleted extracts did not restore MT growth either in interphase or in metaphase (Figure 4A). This indicates that EB1 is not required for MT stabilization in interphase, and that in metaphase, it cannot stabilize MTs in the absence of XMAP215. Moreover, this shows that in metaphase, physiological XMAP215 concentrations cannot support MT growth in the absence of EB1. EB1 depletion did not cause any decrease in the amount of XMAP215 left in the supernatant, and we never found EB1 in XMAP215 IPs or vice versa. This excludes the possibility that the destabilization of MTs observed in EB1-depleted extracts is caused by a co-depletion of XMAP215. Aster size was not affected by APC depletion either in interphase or metaphase extracts (Figure S2B), arguing against the idea that APC stabilizes MTs [18,19] in *Xenopus* egg extracts.

**Figure 5:**
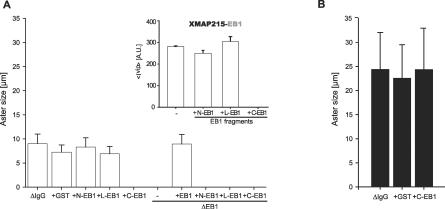
C-EB1 Inhibits XMAP215-EB1 Interaction and Destabilizes MTs in Metaphase *Xenopus* Egg Extracts (A) Effect of EB1 fragments on aster size (±S.D., *n* > 30) in undepleted and ΔEB1 metaphase extracts. VIP experiment of XMAP215/EB1-EGFP interaction in metaphase extract in response to addition of buffer (control), N-terminal domain (N-EB1), linker domain (L-EB1), or C-terminal domain (C-EB1) of EB1 (inset). Error bars represent the s.e.m. of <I_vip_> measured in three different microscopic fields. (B) Effect of C-EB1 on aster size in interphase (± S.D., *n* > 30)

Together with the VIP results, these data strongly suggested a physiological importance for the XMAP215-EB1 interaction in metaphase providing a first indication of a causal relationship between the two MAP network states and the respective MT phenotypes. An interaction between the *Dictyostelium* XMAP215 (DdCP224) and EB1 homologs has been reported using IP [20] and tandem affinity purification [21]. We also observed a direct interaction between purified XMAP215 and EB1 by in vitro VIP and FRET assays (Figure 2C). This shows that both proteins do interact directly, but that this interaction is inhibited in interphase egg extracts (Figure 3A). From the VIP and FRET data and the measured endogenous concentrations of the two proteins (270 nM for EB1 and 600 nM for XMAP215 [4,22] and our unpublished data), we could estimate that at least one third of the EB1 should interact with XMAP215 in metaphase egg extracts. We generated EB1 fragments (Figure S1A) in an attempt to directly interfere with this interaction in the extracts. The C-terminal part of EB1 (C-EB1) turned out to efficiently bind to XMAP215 (Figure 2E), thereby obstructing its interaction with full-length EB1 (Figure 5A, inset). This fragment also completely inhibited MT growth in metaphase just as EB1 depletion did (Figure 5A). By contrast, in interphase extracts, C-EB1 did not induce the formation of short asters (Figure 5B), corroborating the mitotic-specific function of XMAP215-EB1 interaction. Removal of p150^glued^ from metaphase extracts did not have an effect on aster size (Figure S2C), and C-EB1 interacted very weakly with EB1 (Figure 2E, right panel), excluding the possibility that the C-EB1 phenotype was created by interference with p150^glued^-EB1 interaction [23,24] or artificial auto-inhibition of EB1 by C-EB1 [25]. Although we cannot exclude that C-EB1 destabilizes MTs by some unknown mechanism, the data strongly suggest that the specific interaction observed in metaphase between XMAP215 and EB1 is required for proper MT dynamics in this cytoplasmic state.

**Figure 6:**
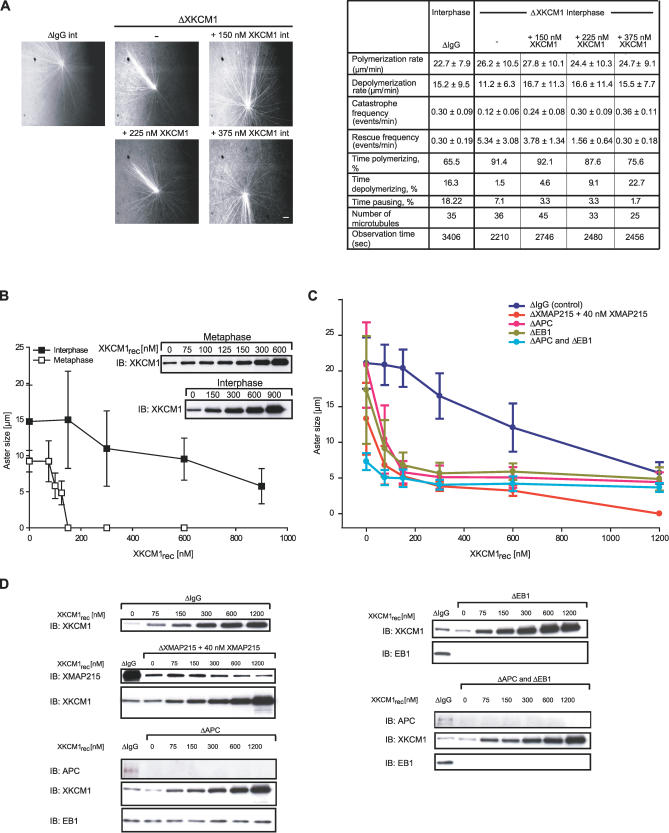
The MT Destabilizing Activity of XKCM1 Is Inhibited by MAPs in Interphase Egg Extracts (A) Still images and table summarizing the dynamic parameters of MT growth in response to XKCM1 depletion (ΔXKCM1) and add back of the recombinant protein. (B) Aster size after stepwise addition of recombinant XKCM1 into undepleted interphase and metaphase extracts. Error bars represent the S.D. (*n* > 30). Inset shows the Western blot of extracts containing increasing amounts of recombinant XKCM1 (XKCM1_rec_ = amount of XKCM1 added in nM; and IB: XKCM1 = immunoblot with anti-XKCM1 antibody). (C) Aster size after stepwise addition of recombinant XKCM1 into control (IgG) depleted (indigo line), XMAP215 depleted plus 40 nM XMAP215 (red line), APC depleted (pink line), EB1 depleted (dark yellow line), and APC and EB1 depleted (cyan line) interphase extracts. Error bars represent the S.D. (*n* > 30). (D) Western blot of extracts containing increasing amounts of recombinant XKCM1 (XKCM1_rec_ = amount of XKCM1 added in nM; and IB: XKCM1 = immunoblot with anti-XKCM1 antibody) that were depleted of specified MAPs.

In interphase, the situation is different: XMAP215 does not interact with EB1, but MTs are long and extremely stable. In this case, the main MT destabilizer, XKCM1, interacts with XMAP215, EB1, CLIP170, and APC. We therefore examined whether the catastrophe-promoting activity of XKCM1 could be inhibited by these interactions. Indeed in interphase extracts, there are so few catastrophes that even when XKCM1 is depleted, there is no significant increase in the size of asters (Figure S2A). The analysis of MT dynamics in interphase extracts depleted from XKCM1 showed marginally significant effects on the catastrophe frequency, growth, and shrinking rates as well as a seemingly large effect on rescue frequency (Figure 6A). The poor effect on catastrophe frequency was expected due to the already very low catastrophe frequency in interphase cytoplasm [12,17], and the apparent increase in rescues is not statistically significant because only three shrinking MTs were observed over a very short shrinking period (f_res_ = rescue events per depolymerization time). This indicated that XKCM1 is inactive in interphase. In order to test this, we added increasing amounts of XKCM1 to interphase and metaphase extracts. In metaphase, the addition of 150 nM XKCM1 above the endogenous concentration (120 nM [5]) completely blocked MT growth, whereas in interphase, the addition of a large excess of XKCM1 (600 nM) did not eliminate MT growth (Figure 6B).

If XKCM1 were inactivated through its interaction with MAPs in the interphase extract, adding it to extracts depleted of individual interaction partners should have a strong effect on MT destabilization. As shown in Figure 6C and 6D, this was indeed the case. We first added increasing amounts of XKCM1 to an extract depleted of XMAP215 and supplemented with a small amount of XMAP215 (40 nM) in order to be able to observe MTs of a significant length (∼13.3 μm). Adding only 75 nM XKCM1 to these extracts dramatically reduced the size of MTs, whereas in the control extract, significant destabilization occurred only around 600 nM (Figure 6C and 6D). Then, we added increasing amounts of XKCM1 to extracts from which we had removed APC or EB1 or both APC and EB1. When APC or EB1 were individually depleted from the interphase extract, XKCM1 strongly destabilized MTs at 75 nM (Figure 6C and 6D). Interestingly, although depleting EB1 or APC alone had no effect on MT length, the double depletion had a dramatic effect, leading to a reduction in MT size from about 20 μm to 7 μm (Figure 6C and 6D). In this case, adding excess XKCM1 lead only to a marginal additional shortening of MTs. As seen in Figure 6C and 6D, adding a large excess of XKCM1 does not fully inhibit MT growth (we observed a minimum length of about 5 μm) in the interphase extract depleted of APC, EB1, or both APC and EB1. The only way to eliminate MT growth was to deplete XMAP215 or to add excess XKCM1 in the extract that was partially depleted of XMAP215 (Figure 4A, Figure 6C and 6D, and Figure S2A).

This indicates that APC and EB1 have a synergistic effect on MT growth in interphase by inactivating XKCM1. To further establish this “buffering” effect of APC and EB1 on XKCM1 activity, we carried out a triple depletion of APC, EB1, and XKCM1 from the interphase extract. If the lack of APC and EB1 caused a shortening of MTs by unleashing XKCM1, removal of endogenous XKCM1 should rescue aster size to wild-type length. This is indeed the case as shown in Figure S2D. These functional data together with our VIP results strongly suggest that in interphase, MTs are stable, because the catastrophe-promoting activity of XKCM1 is inhibited through multiple interactions with XMAP215, APC, and EB1.

## Discussion

We hypothesized that a MAP interaction network may change state when cells alternate between interphase and mitosis, resulting in different MT dynamics. To test this hypothesis, we developed VIP and used it in *Xenopus* egg extracts in which the cytoplasm is unperturbed [26]. Such extracts also support the assembly of nuclei as well as spindles around added DNA in a cell cycle–dependent manner [27].

Here, we have shown that VIP allows the visualization of interactions “in extract” that have been reported to occur by classical IPs. This, together with other controls involving FRET techniques, validates the VIP method. However, VIP has some limitations. The epitopes of some molecules may not be accessible to the antibodies used. It is therefore desirable to check interactions with several antibodies and to include positive controls in the assay whenever possible. VIP does not tell whether the interaction is direct or indirect, and it requires purified recombinant proteins and specific antibodies, as well as fluorescently labeled proteins. However, unlike FRET assays, VIP does not require that both interaction partners be recombinant, fluorescently labeled proteins. Furthermore, multiple interaction pairs can be visualized simultaneously in the same image. The use of VIP can easily be extended to the study of spatial regulation of interaction networks, since interaction signals are stored together with bead coordinates. Most importantly, measurements are made in situ, so that molecular interactions can be monitored in extracts shifting between cell-cycle states.

Using this technique, we have revealed a systematic change in the configuration of MAPs interactions between interphase and mitosis. Most strikingly, a major MT destabilizer, XKCM1, was found to interact with EB1, XMAP215, APC, and CLIP170 in interphase, interactions that are all lost or considerably reduced in the metaphase extract. This change in interactions may be caused by still-unknown phosphorylation events. By contrast, we found a robust interaction between XMAP215 and EB1 in metaphase that could not be detected in the interphase cytoplasm. This is probably also due to phosphorylation changes since this interaction cannot be revealed by classical IP (Figure 2D), which suggests that the phosphorylation state of these molecules is dynamic and lost quickly when the extract is diluted. An interesting future challenge will be to determine what kind of phosphorylation events are required to induce the switch in the overall MAP interaction pattern.

Because VIP does not allow the determination of whether the interactions detected are direct, it is likely that the network of protein interactions involved in the regulation of MT dynamics is larger than the one we have revealed. In effect, the action of the C-EB1 on MT dynamics in metaphase (Figure 5A) may be the result, not only of an inhibition of the XMAP215-EB1 interaction, (Figure 5A, inset), but also of a more complex change in a series of interactions between partners of the network.

We have provided experimental results in support of the idea that there is a causal relationship between the two different states of this small network of interactions and the corresponding state of MT dynamics. In this context, it is interesting that XMAP215 interacts with tubulin both in interphase and mitosis. This suggests that in extracts, MT growth occurs by incorporation of tubulin-XMAP215 complexes. XMAP215 is supposed to stabilize tubulin oligomers and may lead to the incorporation of short protofilament segments into MTs, which would explain its effect on the MT growth rate [28]. In connection with this and in relation to the regulation of MT dynamics between interphase and mitosis, it is interesting to note that in interphase, XMAP215 interacts with XKCM1, which is surrounded by EB1, CLIP170, and APC, whereas in mitosis, it does not interact with XKCM1, but rather with EB1. If we assume that XMAP215 is incorporated into MTs together with tubulin subunits, one could imagine that these other molecules come along as well. In interphase, XKCM1 catastrophe activity seems to be buffered out through its interactions with the other MAPs. It may be incorporated in the growing MT in an inactive form, whereas in metaphase it may be free to act directly at the tip of MTs or diffuse passively along the MT lattice toward the plus end to induce catastrophes [29]. In mitosis, the tubulin-XMAP215-EB1 complex seems important to determine the exact MT dynamics of the mitotic state because removal of EB1 eliminates all MT growth. It is not surprising that an overexpression of XMAP215 overcomes EB1 depletion if we assume that the tubulin-XMAP215 complex is the unit that is incorporated into forming MTs. Indeed, by increasing the concentration of this complex, one increases the concentration of subunits available for MT assembly, thereby favoring it. Another interesting outcome of this study concerns the function of APC and EB1. It has been suggested on the basis of in vitro results that these two molecules stabilize MTs when they interact [6]. Here we found that under physiological conditions, they do not interact—at least not in the cytoplasm. Moreover, EB1 is neither essential for MT growth in interphase nor in mitosis, in which it seems to have a regulatory role. APC is also not required for MT growth either in mitosis or interphase. However, double depletion of APC and EB1 does result in very short MTs in interphase (Figure 6C and 6D). If one looks at the interactions between EB1, XKCM1, and APC in interphase (Figure 3A), this is entirely consistent with the idea that the catastrophe-promoting activity of XKCM1 is inhibited by its interaction with XMAP215, APC, and EB1. Indeed, when both EB1 and APC are depleted, the steady-state concentration of free XKCM1 probably increases, resulting in MT destabilization. We actually demonstrated this by doing a triple depletion experiment of APC, EB1, and XKCM1 from interphase extracts that did not result in MT shortening. On the contrary, upon depletion of XKCM1 in the ΔAPC and ΔEB1 extract, MTs grew from rather short ones (∼9 μm) to almost control levels (∼18 μm), demonstrating that XKCM1 was responsible for MT destabilization in the ΔAPC and ΔEB1 extract. The observation that EB1 or APC single depletion does not significantly destabilize MTs in interphase does not conflict with our model. It rather indicates that there is an excess of “XKCM1 buffering capacity” present in interphase extracts constituted by a variety of MAPs whose individual functions are somewhat interchangeable as far as inactivation of XKCM1 is concerned. This may explain how the inhibition of XKCM1 activity in interphase can be maintained at a constant level even if one specific MAP is removed. Thus, the inhibitory effect of individual MAP depletion on XKCM1 activity only becomes obvious when the “total buffering capacity” is overloaded (e.g., by adding exogenous XKCM1).

It seems that we are getting closer to an understanding of how MT growth is regulated in interphase and mitosis: MT assembly in vivo probably does not only involve tubulin assembly into tubes. Rather, tube assembly may involve oligomeric complexes of tubulin and other MAPs. It is conceivable that XMAP215 has evolved as a major linker and essential protein between tubulin and these other MAPs. Regulation of MT assembly between interphase and metaphase then largely occurs through a regulation of the state of dynamic interactions between XMAP215 and other MAPs in solution. It remains to be determined how this is achieved by CDK1: directly or indirectly. It is tempting to think that CDK1 alters the balance between kinases and phosphatases that modulate the dynamics of interactions between XMAP215 and the other MAPs.

## Materials and Methods

### Antibodies.

Antibodies directed against GST protein as well as against Xenopus laevis full-length EB1, XKCM1 (1–264 amino acids), CLIP170 H2, APCMTBD, and XMAP215 (1–560 amino acids) (Figure S1A) were generated by immunizing rabbits with recombinant proteins according to standard procedures. Polyclonal sera were affinity purified on recombinant proteins covalently linked to NHS-activated Sepharose (Pharmacia, Uppsala, Sweden). Antibody directed against p150^glued^ was purchased from BD Transduction Laboratories (Lexington, Kentucky, United States). Monoclonal mouse-raised antibody directed against GST protein ([m-GST] GST 26H1) was purchased from Cell Signalling Technology (Beverly, Massachusetts, United States).

### Cloning of expression constructs.

MAP cDNAs are from X. laevis and, unless otherwise noted, comprise the complete cDNA sequence. EB1-EGFP was generated by fusing the EGFP (Clontech, Palo Alto, California, United States) cDNA sequence via a linking region (encoding 10xGly) to the sequence of EB1. Both, the EB1 and EGFP-EB1 cDNA sequences were subcloned into a pHAT2 vector for bacterial expression. GST-EB1 fragments were generated by PCR using the EB1 cDNA as a template and inserted into the pGEX 6P-1 expression vector (Pharmacia). The XMAP215 cDNA clone was a gift from A. Popov (INSERM, Grenoble, France). For expression in SF9 cells, XKCM1 cDNA was generated by PCR and placed into pFASTBac I (Invitrogen, Carlsbad, California, United States). EGFP-XKCM1 was created by subcloning of the EGFP and XKCM1 sequences into the pFASTBac HTa vector (Invitrogen). The MT-binding domain of APC (APCMTBD) was generated by PCR of APC as a template and inserted into pGEX-6P-I (Pharmacia).

### Protein expression, purification, and fluorescent labeling.

X. laevis EB1 and GST-EB1 fragments, GST-APCMTBD were expressed in *Escherichia coli* (BL21) and purified on TALON beads (Clontech) and glutathione Sepharose beads (Pharmacia), respectively, according to the manufacturers' instructions. XMAP215 and XKCM1 were expressed and purified as previously described [3,30]. His-EGFP-XKCM1 was expressed in Sf9 cells using the BactoBac system (Invitrogen) following the manufacturer's instructions. The purification was done 48-h postinfection with 50 ml of SF9 cells (1 × 10^6^ cells/ml) expressing X. laevis full-length EGFP-XKCM1 (Figure S1B), which was harvested by centrifugation at 500×*g* for 10 min. Pellets were washed with phosphate buffered saline (PBS) containing a protease inhibitor cocktail (Roche Cat.No.11 873 580 001; Basel, Switzerland) and finally resuspended in 10 ml of PBS + protease inhibitors. Lysis was achieved by three freeze/thaw rounds, and the crude lysate was cleared by a 10-min centrifugation step at 12,000×*g*. The cleared lysate was bound to 0.5 ml of pre-equilibrated TALON beads (Clontech) and incubated at +4 °C for 1.5 h .The resin was washed three times with PBS + 10 mM imidazole, and the protein was eluted in four steps with 0.5 ml of PBS + 150 mM imidazole. The protein concentration was determined, and the buffer was changed to BRB80 (80 mM PIPES [pH6.8], 1 mM MgCl2, and 1 mM EGTA) + 10% glycerol by dialysis.

Fluorescent labeling of proteins was performed using Cy5- and Cy3-monofunctional reactive dye (Amersham Biosciences, Little Chalfont, United Kingdom) according to the manufacturer's instructions. Active CDK1 was purified from *Xenopus* egg extract as previously described [16].

### Extract preparation.

X. laevis cytostatic-factor metaphase-arrested extracts were prepared as described [13]. Interphase extracts were prepared by adding 0.4-mg/ml CaCl_2_ to the metaphase extract and incubated for 90 min at 20 °C. Aliquots of extracts were frozen in liquid ethane, stored in liquid nitrogen, and quickly thawed just before use.

### MAP Immunodepletions, IP, and Western blots.

Extracts were immunodepleted of MAPs as described elsewhere [31] and directly used for spindown of MT asters according to published procedures [32]. For MT dynamics experiments, the extract was depleted prior to freezing in liquid ethane. For evaluation of endogenous and exogenous MAPs levels in the extract, 0.5 μl of the sample was loaded onto either 6%–15% (for EB1, XMAP215, and XKCM1), or 4%–15% (for APC) SDS-PAGE gradient gels and processed for Western blotting. IPs were performed in the same way as immunodepletions for appropriate MAPs. The obtained beads were washed four times with PBS + 0.1% Triton X-100 and dissolved in the sample buffer, and then the entire volume was loaded onto SDS-PAGE gradient gels and processed for Western blotting.

### MT aster size and dynamics.

Images of asters were acquired using a Zeiss Axiovert 135 microscope (Zeiss, Oberkochen, Germany) equipped with a CoolSNAP camera (Roper Scientific, Trenton, New Jersey, United States), a 63× Plan-apochromat NA 1.4 oil-immersion objective lens, and a long-pass rhodamine filter (Chroma Technology Corp, Rockingham, Vermont, United States). The shape of the asters was classified into either radial or comet-like. For the former, an integrated circular intensity profile was generated, whereas for the latter (majority in interphase), an intensity profile along the major axis of the comet was generated. We used a custom macro written using MATLAB (MatWorks) to quantify the average length of MTs in each aster on the basis of profiles representing the average pixel value at radially increasing distances from the aster center. Aster size was determined by the distance corresponding to the half-maximum of the profile amplitude [33]. At least 30 asters per condition were analyzed.

Time-lapse recordings (2-s intervals) were obtained using a Zeiss Axiovert 200 microscope equipped with a CoolSNAP camera (Roper Scientific), a 100× Plan-apochromat NA 1.4 oil-immersion objective lens, and a long-pass rhodamine filter (Chroma). After measuring MT length in the films, MT dynamics were analyzed using a custom-written MATLAB macro to determine the growth rate (V_g_), the shrinkage rate (V_s_), the catastrophe frequency (f_cat_), and the rescue frequency (f_res_) from graphs in which length of MT versus time is plotted. Minimum slope for MT growth or shrinkage was set to 0.05 μm/sec, and minimal growth or shrinkage length since last transition was set to 0.5 μm. MT growth/shrinkage/pause tendency had to persist for at least 6 s in addition to the above-mentioned criteria for a transition event to be scored; otherwise a change in MT behavior would be contributed to a tracking error.

### VIP sample preparation and data acquisition.

Magnetic protein-A (Invitrogen) beads were incubated with saturating amounts of polyclonal, affinity-purified anti-MAP antibody (bait bead) or Alexa405-labeled IgG (control) for 1.5 h at 4 °C. Different baits were encoded by tracing the fraction of specific MAP antibody with different amounts of Alexa405-IgG. The extract was pretreated with 40 μM nocodazole for 20 min on ice to completely inhibit MT polymerization. The nocodazole-treated extract was incubated with fluorescent prey protein, concentrated hemoglobin solution, and a mix of bait and control beads for 15 min at 20 °C. Prey proteins were added at near endogenous concentrations (e.g., XKCM1-EGFP and EB1-EGFP at 500 nM). Each lane of a plastic perfusion chamber (Ibidi, Munich, Germany) was loaded with 20–40 μl of the extract/bead suspension. Images were acquired with a SP2 AOBS laser-scanning microscope (Leica, Wetzlar, Germany) using a HCX 63× Plan-apochromat NA 1.4 oil-immersion objective lens. Alexa405 (detection: 410–470 nm), EGFP (detection: 500–540 nm), and Cy5 emission (detection: 640–730 nm) were acquired by sequential line scanning using the 405-nm, 488-nm, and 633-nm laser lines for excitation. For each sample, at least three different microscopic fields were acquired for calculation of the standard error of the mean (s.e.m.) All routines for automated VIP-image-processing were written in MATLAB 6.1 (flow chart of algorithm: Figure 1B).

### FRET assay of XMAP215-EB1 interaction.

EB1Cy3 (FRET acceptor) was titrated into a 500 nM solution of XMAP215-EGFP (FRET donor) in CSFXB buffer (0.1 M KCl, 2 mM MgCl_2_, 0.1 mM CaCl_2_, and 5 mM EGTA) at 20 °C. Donor emission was measured as a function of EB1Cy3 concentration using a spectrofluorometer (Photon Technology, Lawrenceville, New Jersey, United States) and corrected for dilution. From the quenched donor emission, E_FRET_ was calculated as E_FRET_(A) = (I_A0_ − I_A_)/I_A0_ where I_A_ is the EGFP emission in the presence of acceptor and I_A0_ is the EGFP emission without acceptor. The binding isotherm (A → E_FRET_) was fitted with a one site–binding model to obtain the Kd of the interaction.

## Supplementary Information

Figure S1 Constructs and Recombinant Proteins Used (A) List of (labeled) recombinant proteins/protein fragments used (left panel) and list of affinity purified antibodies used (right panel).
(B) Baculovirus expression and purification of XKCM1-EGFP. Coomassie blue–stained gel of crude Sf9 cells lysate, cleared Sf9 cells lysate, flow through, and eluted XKCM1-EGFP.
(1.5 MB PDF)

Figure S2 Combined Effects of MAPs Depletion of Aster Size (A) Impact of XMAP215 and XKCM1 depletion (ΔXMAP215/ΔXKCM1) on aster size. Upper panel: immunoblot (IB) of MAPs after indicated treatment.
(B) Impact of APC and EB1 depletion (ΔAPC/ΔEB1) on aster size. Upper panel: immunoblot (IB) of MAPs after indicated treatment.
(C) Impact of p150^glued^ depletion on aster size in metaphase and interphase extracts.
(D) Impact of APC and EB1 depletion (ΔAPC&ΔEB1) as well as of APC and EB1 and XKCM1 depletion (ΔAPC&ΔEB1&ΔXKCM1) on aster size in interphase extract. Upper panel: immunoblot (IB) of MAPs after indicated treatment. Error bars represent the standard deviation (S.D.; *n* > 30).
(3.4 MB PDF)

## Abbreviations

APCMTBD: APC-microtubule binding domain
CDK1: cyclin-dependent kinase 1
C-EB1: C-terminal domain of EB1
EGFP: enhanced green fluorescent protein
FRET: fluorescence resonance energy transfer
IP: immunoprecipitation
<Ivip>: the median of the specific bait-bead intensities
MAP: microtubule-associated protein
MT: microtubule
PBS: phosphate buffered saline
S.D.: standard deviation
s.e.m.: standard error of the mean
VIP: visual immunoprecipitation
