# Supplementary material for: Discrete States of a Protein Interaction Network Govern Interphase and Mitotic Microtubule Dynamics
Source: PLoS Biol. 2007 Jan 16;5(2):e29. doi: 10.1371/journal.pbio.0050029 (PMC1769425; doi:10.1371/journal.pbio.0050029)

Figure S1

A

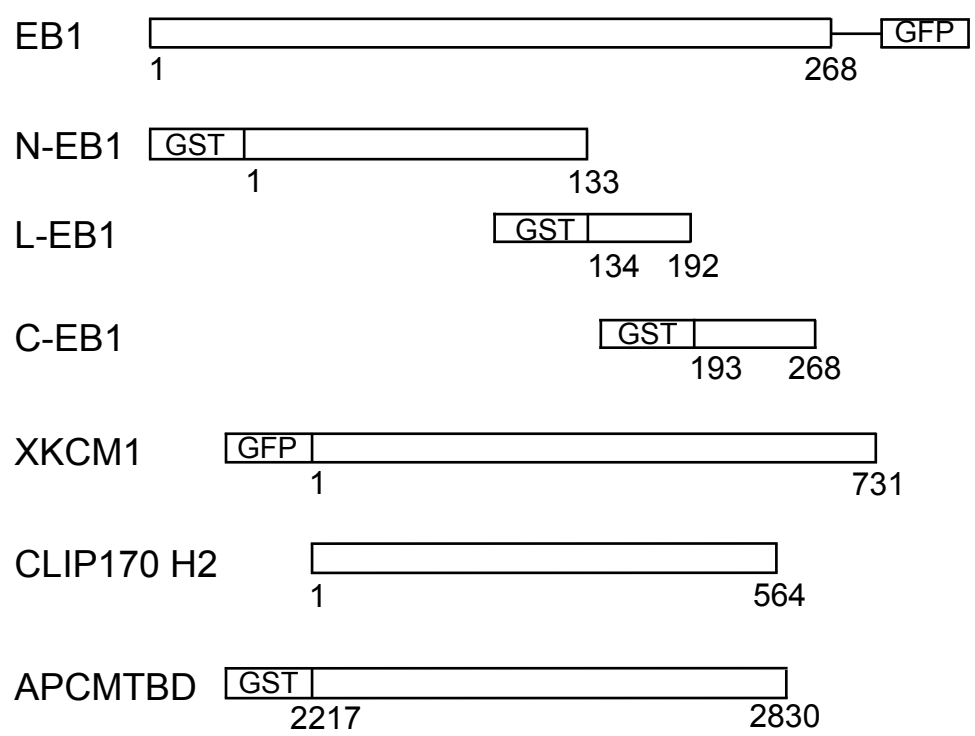

Antibodies	raised against
APC	Microtubule Binding domain (2217aa-2830aa)
EB1	full length Xenopus laevis protein
CLIP-170	H2 domain (1aa-564aa)
XKCM1	fragment (1aa-264aa)
XMAP215	fragment (1aa-560aa)
GST	GST protein

B

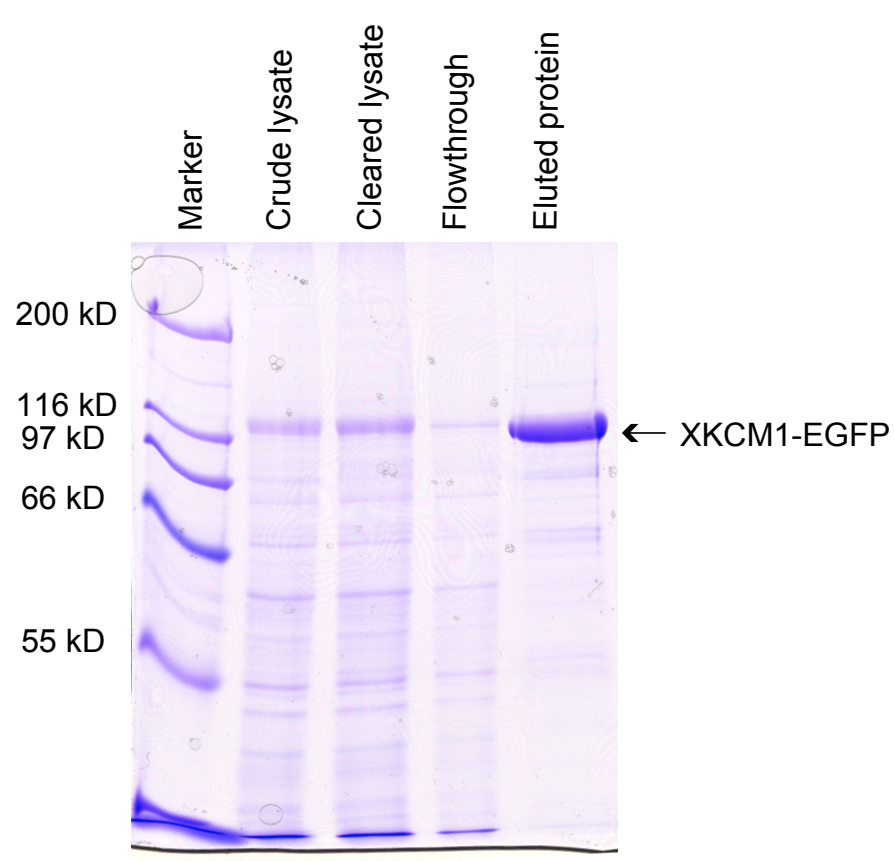

Supplement: Supplementary file 1 [file pbio.0050029.sg001.pdf]
