# Supplementary material for: Discrete States of a Protein Interaction Network Govern Interphase and Mitotic Microtubule Dynamics
Source: PLoS Biol. 2007 Jan 16;5(2):e29. doi: 10.1371/journal.pbio.0050029 (PMC1769425; doi:10.1371/journal.pbio.0050029)
Supplement: Supplementary file 2 [file pbio.0050029.sg002.pdf]

Figure S2

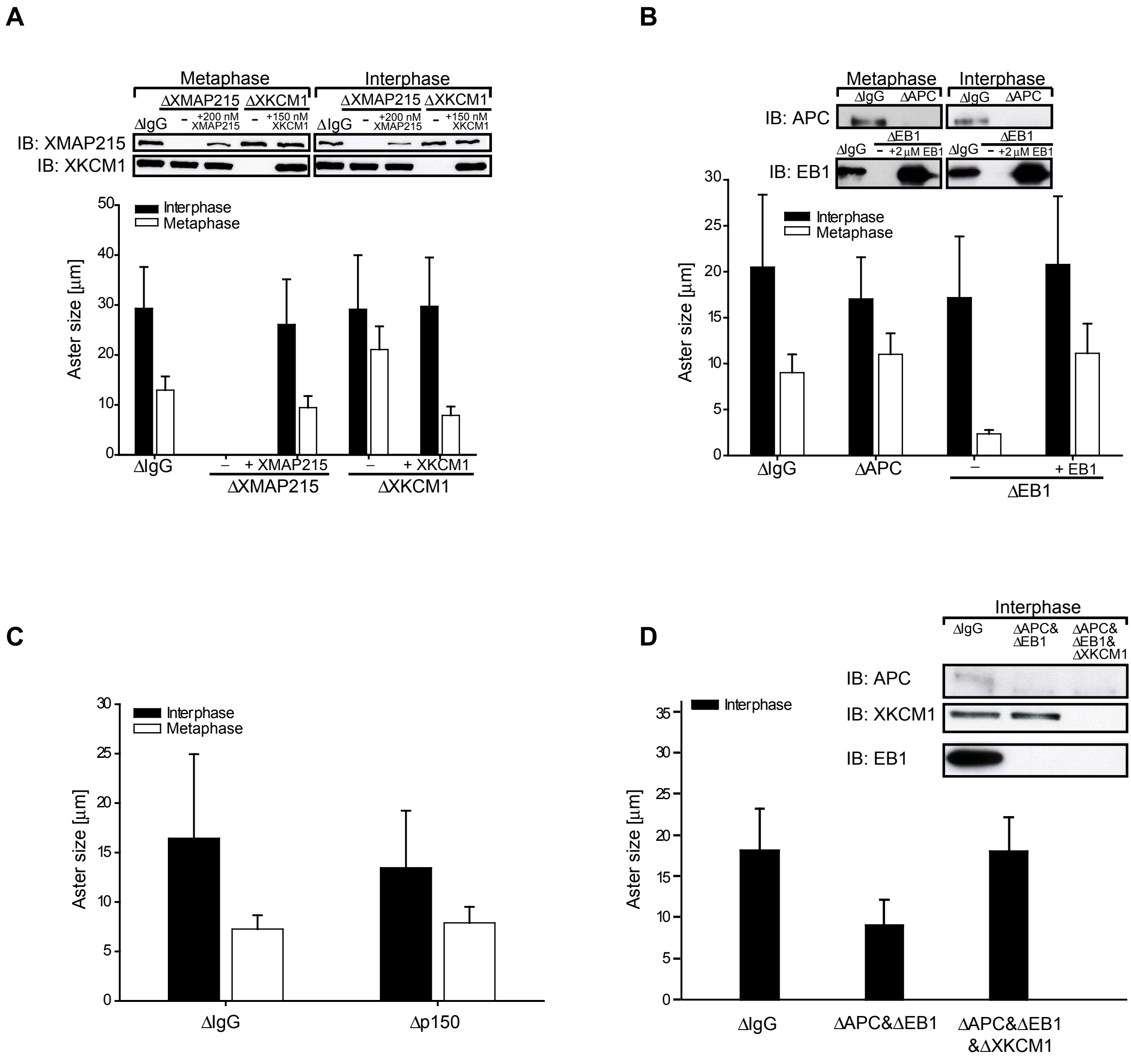

A
Metaphase
Interphase
ΔXMAP215
ΔXKCM1
ΔIgG
+200 nM XMAP215
+150 nM XKCM1
IB: XMAP215
IB: XKCM1
Interphase
Metaphase
Aster size [μm]
ΔIgG
+ XMAP215
+ XKCM1
ΔXMAP215
ΔXKCM1
B
Metaphase
Interphase
ΔIgG
ΔAPC
IB: APC
ΔEB1
+2 μM EB1
IB: EB1
Interphase
Metaphase
Aster size [μm]
ΔIgG
ΔAPC
+ EB1
ΔEB1
C
Interphase
Metaphase
Aster size [μm]
ΔIgG
Δp150
D
Interphase
ΔIgG
ΔAPC& ΔEB1
ΔAPC& ΔEB1& ΔXKCM1
IB: APC
IB: XKCM1
IB: EB1
Interphase
Aster size [μm]
ΔIgG
ΔAPC&ΔEB1
ΔAPC&ΔEB1 &ΔXKCM1
